# Proteomic Analysis of Ovarian Cancer Cells Reveals Dynamic Processes of Protein Secretion and Shedding of Extra-Cellular Domains

**DOI:** 10.1371/journal.pone.0002425

**Published:** 2008-06-18

**Authors:** Vitor M. Faça, Aviva P. Ventura, Mathew P. Fitzgibbon, Sandra R. Pereira-Faça, Sharon J. Pitteri, Ann E. Green, Renee C. Ireton, Qing Zhang, Hong Wang, Kathy C. O'Briant, Charles W. Drescher, Michèl Schummer, Martin W. McIntosh, Beatrice S. Knudsen, Samir M. Hanash

**Affiliations:** Fred Hutchinson Cancer Research Center, Seattle, Washington, United States of America; University of Munich and Center of Integrated Protein Science, Germany

## Abstract

**Background:**

Elucidation of the repertoire of secreted and cell surface proteins of tumor cells is relevant to molecular diagnostics, tumor imaging and targeted therapies. We have characterized the cell surface proteome and the proteins released into the extra-cellular milieu of three ovarian cancer cell lines, CaOV3, OVCAR3 and ES2 and of ovarian tumor cells enriched from ascites fluid.

**Methodology and Findings:**

To differentiate proteins released into the media from protein constituents of media utilized for culture, cells were grown in the presence of [^13^C]-labeled lysine. A biotinylation-based approach was used to capture cell surface associated proteins. Our general experimental strategy consisted of fractionation of proteins from individual compartments followed by proteolytic digestion and LC-MS/MS analysis. In total, some 6,400 proteins were identified with high confidence across all specimens and fractions.

**Conclusions and Significance:**

Protein profiles of the cell lines had substantial similarity to the profiles of human ovarian cancer cells from ascites fluid and included protein markers known to be associated with ovarian cancer. Proteomic analysis indicated extensive shedding from extra-cellular domains of proteins expressed on the cell surface, and remarkably high secretion rates for some proteins (nanograms per million cells per hour). Cell surface and secreted proteins identified by in-depth proteomic profiling of ovarian cancer cells may provide new targets for diagnosis and therapy.

## Introduction

Ovarian cancer is one of the most aggressive and lethal epithelial cancers in women [1]. There are four major histological types of epithelial ovarian cancer: serous, endometrioid, clear cell, and mucinous, which differ in both their clinical behavior and molecular characteristics. The serous subtype is the most commonly diagnosed and is responsible for most ovarian cancer deaths [1]. Currently there is no accurate non-invasive diagnostic test for ovarian cancer; most patients are diagnosed at an advanced stage [2], presenting with metastases and invasion of the peritoneal cavity and ascites [3]. Therefore, the identification of proteins that are abundantly and predominantly expressed in ovarian cancer presents a valuable undertaking and may provide early clues to the presence of ovarian cancer, and/or indications of a molecular subtype that could help to guide treatment.

Identification of the repertoire of proteins that are cleaved from the cell surface and of proteins that are otherwise released into the extracellular compartment can contribute to our understanding of tumor behavior, to the identification of potential diagnostic markers detectable in circulation and of potential imaging and therapeutic targets that remain displayed on the cell surface. During the metastasis of ovarian cancer, the production of matrix-degrading proteinases by tumor cells contributes to the disruption of cell interactions through a mechanism of shedding of adhesion molecules. These mechanisms have been demonstrated in ovarian cancer context for ALCAM [4] and for epithelial cadherin (CDH1)[5].

In-depth, quantitative proteomics allows this delineation of proteins expressed in tumor cells and in sub-cellular compartments [6]. Several proteomic studies have analyzed ovarian tumor tissue [7], [8], cell lines [9]–[11], ascites fluid [12] and blood from patients with ovarian cancer [13], [14]. However, there is still a need to systematically characterize and compare sets of proteins whose locations make them especially relevant to diagnosis and treatment, notably proteins at the cell surface or those released into the extra-cellular milieu, and to determine the mechanism and dynamics of protein release into the extracellular space.

We characterized three ovarian adenocarcinoma cell lines, OVCAR3, CaOV3 and ES2, as well as ovarian cancer cells enriched from ascites fluid with an in-depth proteomic analysis of whole cell lysates, the cell surface proteome and proteins released into the extra-cellular compartment. Detailed exploration of the data revealed several interesting biological phenomena, including extensive shedding of extra-cellular domains for proteins expressed on the cell surface and high secretion rates of a subset of proteins. The data also includes a number of protein markers known to be associated with ovarian cancer. The resulting public dataset is a resource for discovery of diagnostic and therapeutic targets and a rich source of information regarding the distribution of proteins between the cell interior, the cell surface and the extracellular space.

## Methods

### Culture and isotopic labeling of ovarian cancer cells

OVCAR3, CaOV3 and ES2 cells were grown in DMEM media (Invitrogen) containing 0.1% of dialyzed fetal bovine serum (FBS) (Invitrogen) and ^13^C-lysine instead of regular lysine, for 7 passages (1∶2) according the standard SILAC protocol [15]. Incorporation of ^13^C Lys isotope exceeded 90% of the total protein lysine content. The same batch of cells was used for extracting cell surface proteins and for analysis of conditioned media and whole cell lysate proteins. The secreted proteins were obtained directly from the cell conditioned media after 48 h of culture. Cells and debris were removed by centrifugation at 5000×g and filtration through a 0.22 μm filter. Total extracts of cells were obtained by sonication of ∼2×10^7^ cells in 1 ml of PBS containing the detergent octyl-glucoside (OG) (1% w/v) and protease inhibitors (complete protease inhibitor cocktail, Roche Diagnostics, Germany) followed by centrifugation at 20,000×g.

Tumor cells were obtained from 2.2 l of ascites fluid from one patient with ovarian serous adenocarcinoma. Ascites samples were collected under an IRB approved protocol. After centrifugation at 2000 rpm for 5 min, the pellet of cells was washed 3 times with PBS followed by centrifugation at 2000 rpm for 10 min. A gradient of percoll (30 to 60%) was used to enrich the preparation in cells derived from tumors and eliminate contaminant mononuclear blood cells. Tumor cells comprised ∼80% of viable cells in the resulting sample, as evaluated by microscopic inspection. The ascites cell conditioned media was obtained after 24 h of culture in 0.1% of bovine serum albumin (BSA), without addition of fetal bovine serum (FBS). Cells and debris were removed by centrifugation at 5000×g and filtration through a 0.22 μm filter.

### Capture of cell surface proteins

To isolate cell surface proteins from the three adherent ovarian cancer cell lines, ∼2×10^8^ cells were biotinylated in the culture plate after extensive PBS rinsing, with 10 ml of 0.25 mg/ml of Sulfo-NHS-SS-BIOTIN in PBS at room temperature (23–24°C) for 10 min. The residual biotinylation reagent was quenched with 10mM Lysine. Enriched tumor cells from ascites were washed three times in PBS and biotinylated in suspension (2.5×10^8^cells/ml) with 0.48mg/ml of Sulfo-NHS-SS-BIOTIN in PBS for 30 min at room temperature (23–24°C). The residual biotinylation reagent was quenched with 10mM Lysine in both cell line and ascites preparation. Protein extraction was performed in a solution containing NP 40 detergent 2% (v/v) with cell disruption by sonication followed by centrifugation at 20,000×g. Biotinylated proteins were chromatographically isolated by affinity chromatography using 1 ml of UltraLink Immobilized Neutravidin (Pierce) according to manufacturer instruction. Proteins bound to the column were recovered by reduction of the biotinylation reagent with 5 ml of a solution containing 65 μmol of DTT and 1% octyl-glucoside (OG) detergent for 1 h at 37°C. Eluted proteins were subsequently alkylated with 200 μmol of iodoacetamide at room temperature.

### Fractionation of cell extracts

Cell surface, conditioned media and total extract were fractionated by reversed-phase chromatography, using respectively 500 μg, 1 mg and 1 mg of total protein. All the cell extracts were reduced and alkylated with iodoacetamide prior to chromatography. Separation was performed in a POROS R1/10 column (Applied Biosystems–4.6×50 mm) at 2.7 ml/min using a linear gradient of 10 to 80% of organic solvent over 30 minutes run. Solvent system used was: aqueous solvent–5% acentonitrile/95% water/0.1% of trifluoracetic acid; organic solvent-75% acetonitrile/15% isopropanol/10% water/0.095% trifluoracetic acid. Fractions were collected at a rate of 3 fractions/minute.

### Protein identification by LC-MS/MS

Protein digestion and identification by LC-MS/MS was performed as described previously [16]. Briefly, each one of the reversed-phase fractions was individually digested in-solution digestion with trypsin (400 ng/fraction), and grouped into 15 to 21 pools for each cell line and each compartment (i.e. cell surface, conditioned media and soluble whole cell lysate) based on chromatographic features. Pools were individually analyzed by LC-MS/MS in a LTQ-ORBITRAP mass spectrometer (Thermo-Finnigan) coupled to a nanoflow chromatography system (Eksigent) using a 25 cm column (Picofrit 75 μm ID, New Objectives, packed in-house with MagicC18 resin) over a 90 minute linear gradient. Acquired data was automatically processed by the Computational Proteomics Analysis System–CPAS [17]. The tandem mass spectra were searched against version 3.13 of the human IPI database. A fixed modification of 6.020129 mass units was added to lysine residues for database searching to account for incorporation of the heavy lysine isotope. To estimate the significance of peptide and protein matches, we applied the tools PeptideProphet [18] and ProteinProphet [19]. Identifications with a PeptideProphet probability greater than 0.75 were selected and submitted to ProteinProphet. The latter infers a minimal set of proteins that explain the peptide evidence, assigning a probability to each protein based on the combined peptide probabilities. The derived protein identifications were filtered at a 1% error rate based on the probability that the best match obtained would fall in the distribution of random database matches [20]. The spectral counting method [21] was used to estimate protein enrichment for each compartment. The total number of spectral counts for each protein group output by ProteinProphet was used for the semi-quantitative analysis. Each dataset was normalized by the total number of counts in the entire experiment. The enrichment factor was calculated by the following expression: [(C_x p_/Nf)+1]/(C_te p_+1), where C_x_ represents counts of each protein (p) in the sub-proteome (secretion or cell surface); Nf is the normalization factor (presented in the top of Table S1); C_te_ is the counts for the same protein (p) observed in the total extract of the respective cell. The addition of 1 to the counts was intended to take into account those proteins for which no observation was made in one of the sub-proteomes and represents the minimum enrichment factor for that particular protein.

## Results

### Proteomic Analysis of Ovarian Cancer Cells

**Figure 1:**
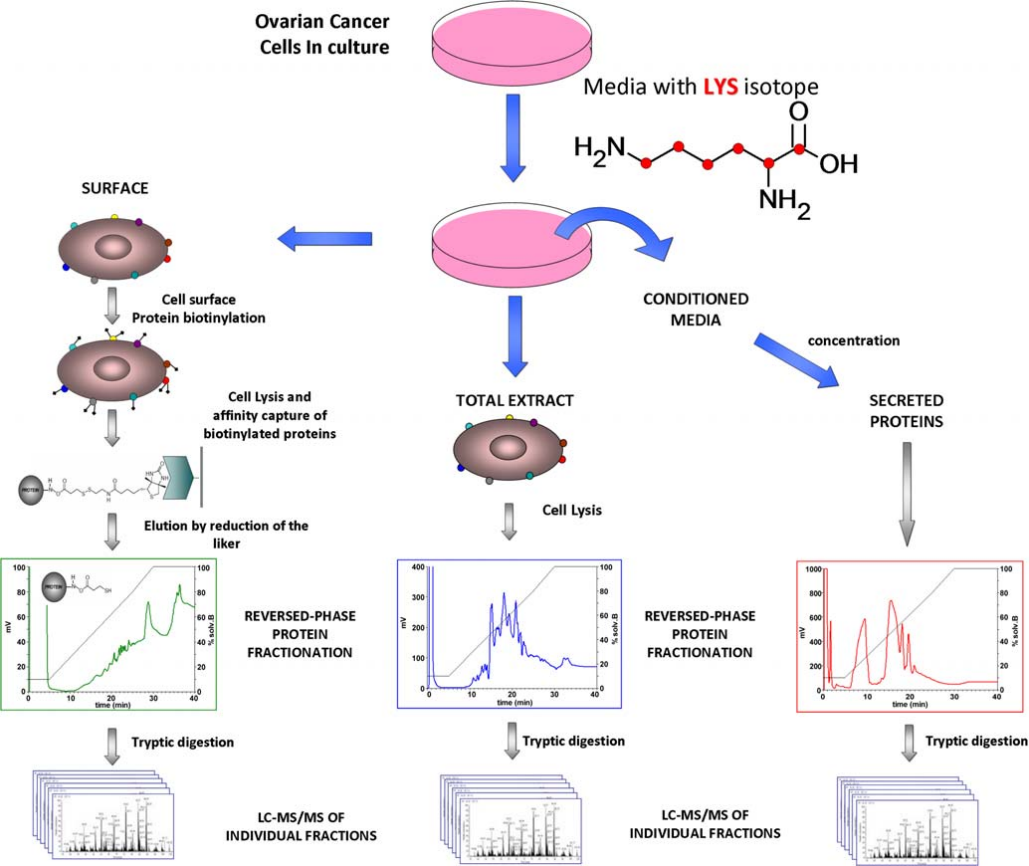
Experimental overview of the analysis of whole cell lysate, cell surface proteins and proteins from the conditioned media of ovarian cell lines and tumor cells derived from ascites.

A comprehensive proteomic profile of the OVCAR3, CaOV3 and ES2 cell lines, and ovarian cancer cells from ascites of a patient with serous ovarian cancer was performed. The general experimental workflow consisted of intact protein fractionation followed by mass spectrometric data acquisition by LC-MS/MS and data analysis, as described in Figure 1. Overall, we performed 215 LC-MS/MS runs, which correspond to more than two million MS scans.

#### Total cell lysates

**Figure 2:**
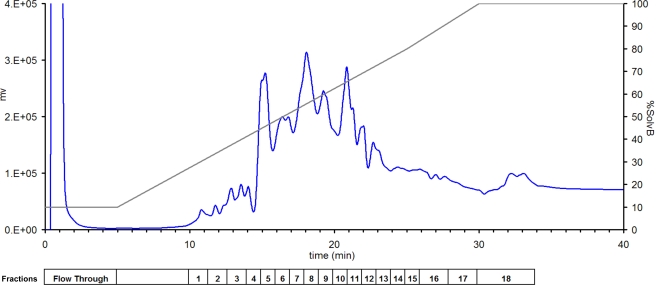
Reversed-phase fractionation of the total extract of ES2 cell line. The chromatogram shows the profile of fractionation of 1 mg of whole cell lysate. We collected 18 fractions of this sample for further protein identification by LC-MS/MS. The decomplexing of samples prior LC-MS/MS analysis by intact protein fractionation improves significantly the identification of low abundance proteins [16]. The boxes under the chromatographic profile indicated the fractions analyzed. The solid dark line indicates the gradient used in the separation.

**Figure 3:**
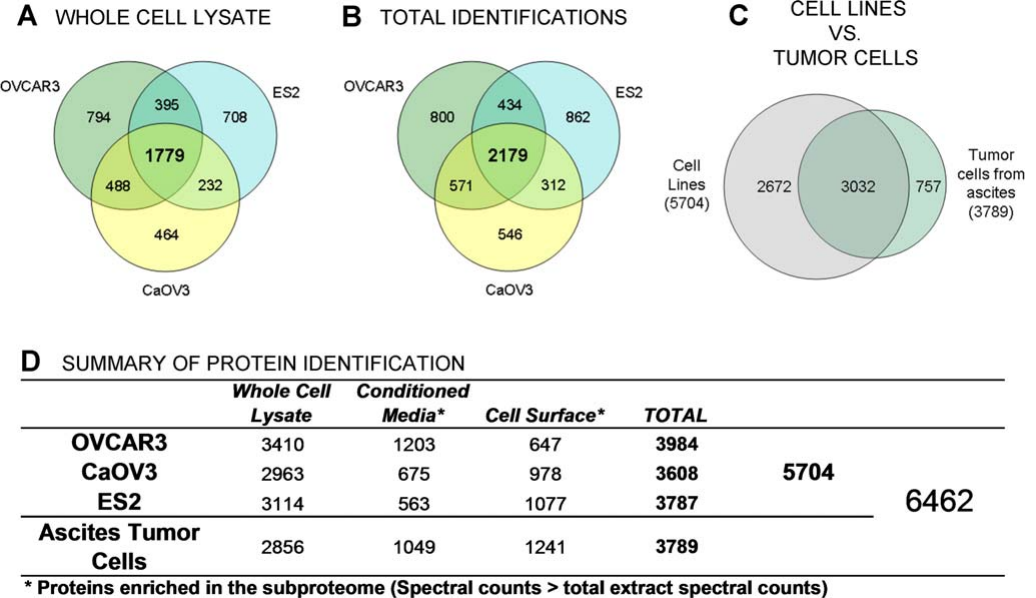
Protein identifications in ovarian cancer cells. Venn diagrams show overlap between proteins identified in different cell populations analyzed. A. Identifications from whole cell lysates of the three cell lines analyzed. B. Proteins identified in the three sub-proteomes (whole cell lysate, conditioned media and cell surface). C. Proteins identified in tumor cells enriched from ascites exhibiting 80% concordance with proteins identified in the three ovarian cancer cell lines. D. Number of proteins identified in each dataset. Table S1 lists the proteins identified in this study.

A reversed-phase chromatogram representing the fractionation of intact proteins in the whole cell lysate of ES2 cells is presented in Figure 2. Overall, the analysis yielded some 3,000 high confidence protein identifications for each cell population analyzed (Figure 3). The number of protein identifications from total cell extracts in common between the three cell lines was 1179, and ranged from 2011 to 2267 for two cell lines (Figure 3A). On average, 655 proteins were uniquely identified in one cell line. In addition to biological heterogeneity, part of the variability in identified proteins between cell lines could be attributed to mass spectrometric undersampling of low abundance proteins.

#### Cell surface proteins

We relied on a labeling strategy with lipid impermeable biotin to tag and capture proteins exposed on the cell surface of OVACAR3, CaOV3, ES2 cell lines and ascites derived tumor cells (Figure 1). From approximately 2×10^8^ cells, we obtained some 500 μg of biotinylated proteins, representing ∼1–2% of the total cell protein mass. The list of protein identifications is presented in Table S1.

#### Proteins released into the medium

The cell lines were grown in culture media containing heavy isotopes of lysine. Isotopic labeling of proteins was intended to discriminate between proteins released from growing cells and proteins intrinsic to supplemented media. After 48 h of culture, the concentration of proteins increased from 100 μg/ml to 225 μg/ml in the conditioned medium of OVCAR3 cells. Because ∼15% of predicted human tryptic peptides with more than 6 amino acids are identical between human proteins and their bovine counterparts, isotopic labeling of cell proteins provided a means to differentiate human proteins secreted by cultured cells from bovine proteins present in the medium. For ascites derived cells, the media were collected after 24 h of culture in the presence of 0.1% bovine serum albumin (BSA). Approximately 1 mg of proteins from the conditioned media from cell lines and cancer cells from ascites were fractionated using the same reversed-phase chromatography method as used for whole cell lysates, and individual fractions were analyzed by LC-MS/MS. For cultured cell lines, proteins that were identified exclusively from peptides without labeled lysine residues and that were identical in sequence between human and bovine, were attributed to culture medium and were not included in the list of proteins released from cells.

The proteomic profile of ovarian cancer cells lead to the identification of 6,462 proteins (Figure 3D). We used strict criteria for protein identifications, considering only valid those proteins with minimum ProteinProphet probability threshold of 0.9. For this threshold, the estimated false identification rate is less than 1%. This dataset represents the most detailed and comprehensive proteomic study of cell populations for ovarian cancer. The depth of analysis and coverage achieved at the protein level in this study is equivalent to the depth of analysis to uncover expressed genes at the RNA level.

### Protein distribution between the intracellular, cell surface and extracellular compartments

A comparative analysis of proteins in different compartments allows assessment of the extent of enrichment of proteins on the cell surface and in the extracellular compartment. We relied on the spectral counting method [21] to estimate protein enrichment for each compartment. After normalization to the total number of spectral counts, proteins with greater number of counts in a compartment (cell surface or conditioned medium) compared to total cell lysates were considered enriched in that compartment (Figure 3). The corresponding enrichment factors observed for all proteins identified is presented in Table S1.

**Figure 4:**
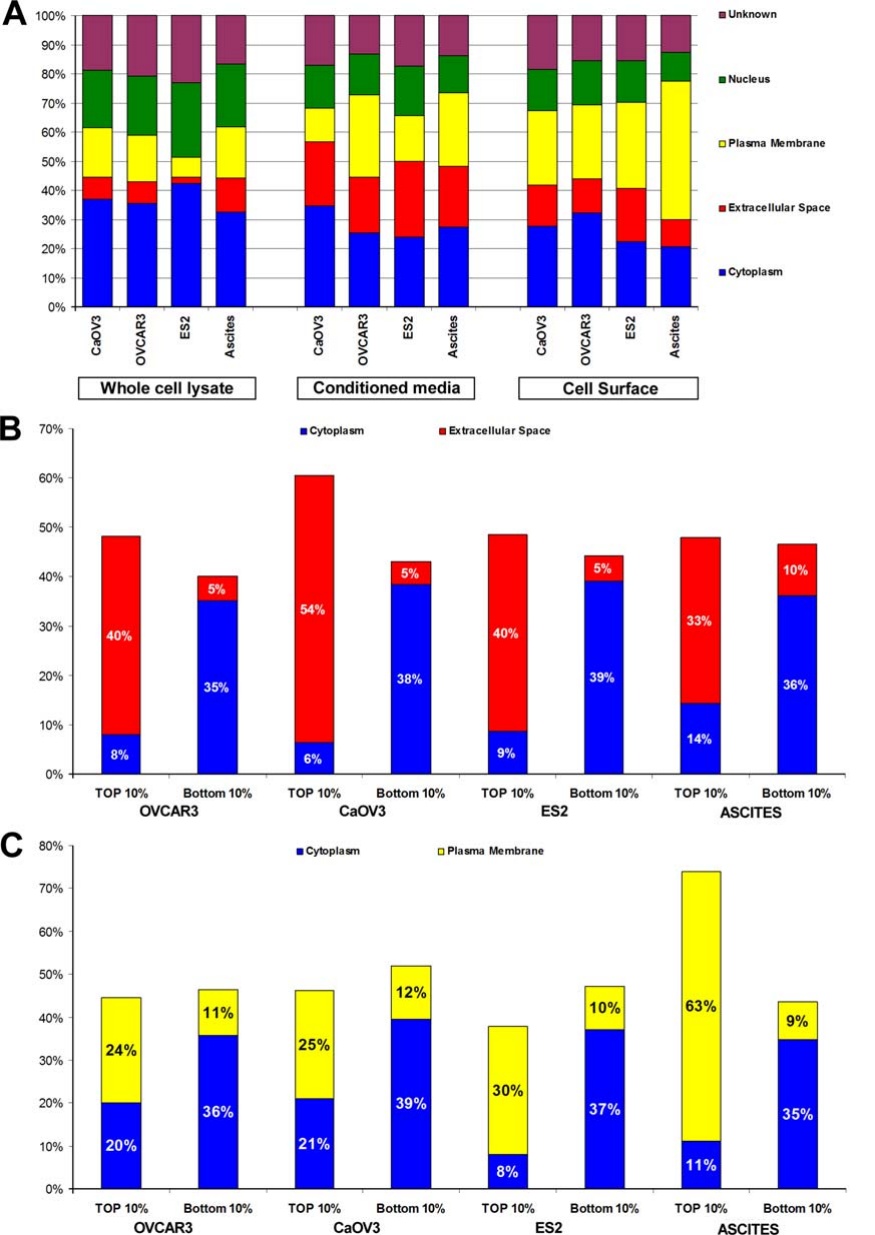
Distribution by cellular location of proteins identified in ovarian cancer cells. A. The cellular localization classification used a combination of annotations from Ingenuity Pathway Analysis software and computational prediction of transmembrane alpha-helix (TMHMM) and signal peptides (SignalP). B. and C. Comparison of the top 10% enriched proteins in conditioned media or cell surface (B and C, respectively) with the least enriched 10% demonstrating strong concordance between predicted cellular location and the specific sub-proteome analyzed.

Proteins attributed to the three cellular compartments were analyzed with the Ingenuity Pathways Analysis (Ingenuity® Systems, www.ingenuity.com), and with algorithms for prediction of transmembrane alpha-helix domains (TMHMM)[22] and for the presence of signal peptides (SignalP)[23] (Figure 4A). This analysis showed substantial concordance between demonstrated location based on our proteomic profile and predictions from database analysis. This concordance was particularly evident for the top 10% enriched proteins when contrasted with the 10% least enriched proteins (Figure 4B and 4C), supporting the strategy utilized to analyze specific compartments. The significant number of proteins annotated as nuclear or cytoplasmic and present among the identifications in both the conditioned media and cell surface sub-proteomes is noteworthy. To some extent, occurrence of these predominantly intracellular proteins may be due to cell lysis since around 1% of cells in culture were estimated to be dead based on trypan blue staining. However, there is prior evidence for the occurrence of cytoplasmic proteins bound to the extracellular membrane, particularly in cancer [24], [25].

Proteins secreted by the cells are expected to diffuse through tissues and enter the circulation. Therefore, in contrast to intracellular proteins, secreted proteins may be more likely detectable in plasma. We compared ovarian cell protein profiles by compartment with proteins listed in the Human Plasma Peptide Atlas [26]. Of the total 6,462 proteins identified in this study, 2,341 (36%) occurred in the Human Plasma Peptide Atlas (Table S1). The top 100 proteins that were enriched in conditioned media for each of the cell populations analyzed were more highly represented in the Peptide Atlas (65 to 78%) compared to the overall 36% representation. These results suggest that secreted proteins are more abundant in plasma that non-secreted proteins.

### Similarities between cell lines and ascites tumor cells

The three cell lines in this study represent well characterized *in vitro* models for ovarian cancer. OVCAR3 and CaOV3 are derived from human serous ovarian adenocarcinoma [27], [28] and ES2 is derived from clear cell carcinoma [29]. Differences in histology are paralleled by differences in protein profiles, as reflected in the clustering analysis using the spectral counts as measures of protein abundance (Figure S1). This is evident from observations that the secreted and surface sub-proteomes of ES2 are significantly different from those of the two other cell lines analyzed.

The tumor cells enriched from ascites we analyzed were derived from a patient with serous ovarian adenocarcinoma. Eighty percent of the proteins identified in the ascites tumor cells were also identified in one or more of the cell lines (Figure 3C). As indicated in Figure S1, patterns for the patient's tumor cells isolated from ascites have greater similarity with CaOV3 and OVCAR3 than with ES2.

Another way to account for similarities can rely on observation of proteins already known to be associated with the disease. Several kallikreins have been previously associated with serous ovarian adenocarcinoma [30]. KLK6, KLK7 and KLK 9 were abundant in the conditioned medium from ascites derived tumor cells, OVCAR3 and CaOV3, but not in ES2 cell conditioned media (Table S2). These observations suggest again greater similarities among the serous adeonocarcinoma cell lines OVCAR3 and CaOV3 and the tumor derived cells as well as the differences in relation to clear cell derived ES2 cell line.

Some differences were observed between ascites derived cells and cell lines. Some proteins were exclusively detected and enriched on the surface or in culture media of ascites derived tumor cells. These include proteins such as ABP1 (amiloride-sensitive amine oxidase) and MMP7 (and matrix mettaloprotease 7), the transcripts for which are notably abundant in various sub-types of ovarian cancer, and others encoded by mRNAs with high expression levels in specific histological types of ovarian cancer (Table S3). Of note is the fact that several of the proteins enriched exclusively in tumor cells from ascites have very low tissue expression in normal ovary [31] (Table S3). These proteins are possibly derived from leukocytes and mesothelial cells that would have been co-purified from ascites fluid.

### Shedding of surface membrane proteins into the extra-cellular compartment

**Figure 5:**
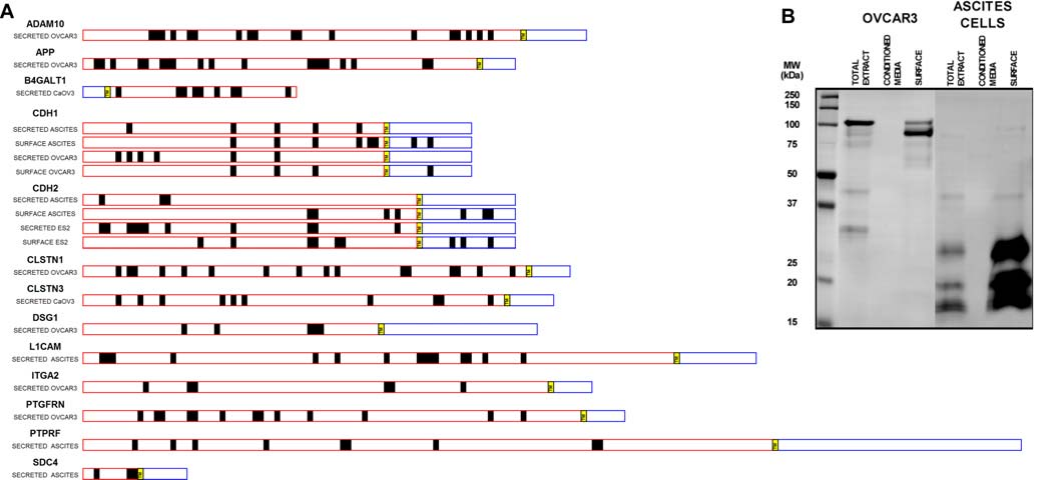
Shedding of membrane proteins from ovarian cancer cells into conditioned media. A. Identification of peptides spanning extracellular domains of transmembrane proteins detected in the conditioned media, consistent with a shedding process. CDH1 (epithelial cadherin) illustrates well this process, since peptides spanning only the extracellular domain are observed in conditioned media while peptides spanning both intracellular and extracellular regions were detected on the surface of Ovcar3 and ascites cells. Red traces indicate extracellular domains and blue traces indicate intracellular domains. Transmembrane regions are indicated in the yellow boxes. Dark boxes indicate peptides identified. B. Western blotting of CDH1 using a specific antibody against the intracellular domain (mouse anti-E-cadherin clone 36, #610181, BD Biosciences) demonstrating cleavage of the full length CDH1. There were no peptides derived from the intracellular domain in conditioned media.

In support of our experimental and analytic approach, we found that a substantial proportion of proteins annotated as membrane proteins were identified in culture medium, representing more than 25% of all identified proteins as for example in OVCAR3 and ascites derived tumor cells (Figure 4a). Several of these proteins were significantly enriched both on the cell surface and in the culture medium (Table S4). Analysis of peptides identified indicated that for the most part, these peptides were derived from extracellular domains of cell surface proteins as shown in Figure 5A, suggesting ectodomain shedding.

Epithelial cadherin (CDH1) clearly illustrates the shedding process, since sequence coverage of the extracellular domain is predominant in the secreted fraction, whereas peptide coverage spanning both intra and extra cellular domains was observed for the protein isolated from the cell surface fraction. We compared the mass spectrometry based findings with western blot analysis of ovarian cell line and ascites-derived tumor cells. Intact forms of CDH1 were observed in whole cell lysates, together with lower molecular fragments containing the intracellular domain. However, in ascites cells there is a predominance of low molecular weight fragments containing only the intracellular domain both in total cell extract and cell surface, indicating that the shedding process is more extensive in tumor cells than in cell lines. Forms of CDH1 containing the intracellular domain were not detected in the conditioned media of these cells (Figure 5b). The process of shedding of CDH1 has recently been described for ovarian cancer cells [5], in support of our mass spectrometry based investigations. Of note is the observation in our study of additional proteins from the cadherin family of adhesion molecules in conditioned media, such as neural cadherin (CDH2), calsyntenin-1 (CLSTN1), alcadein beta (CLSTN3), desmoglein-1 (DSG1) and desmoglein-2 (DSG2). Based on Ingenuity Pathway Analysis annotation, most of these membrane proteins identified enriched in the conditioned media are related to the processes of cell adhesion and cell movement (Table S4). The process of cleavage and shedding is not uniform for all proteins as we have observed a significant number of proteins enriched in the cell surface fraction that are not detected in the cell culture medium (Table S4).

### Protein secretion rates

Analyzing proteins released into the medium by cultured cells is an attractive strategy to identify potential circulating markers. The potential for detection of increased levels of a protein in circulation is dependent in part on the rate at which the protein is released into the extracellular space and ultimately the blood compartment by the tumor relative to other tissues. In the list of proteins most enriched in culture media relative to whole cell lysates were two tissue metalloprotease inhibitors (TIMP 1 and TIMP2) (Table S2). These proteins have direct diagnostics potential in ovarian cancer [32].

**Figure 6:**
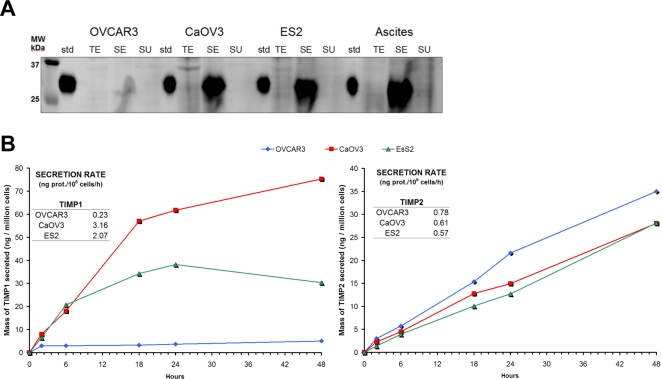
Detection of TIMP 1 and TIMP 2 in the conditioned media of ovarian cancer cells. A. The Western blot shows the presence of TIMP1 only in the conditioned media of all the 4 ovarian cancer cells. OVCAR3 expressed significantly less TIMP1 in comparison to the other cells. Same mass of protein (5μg) was loaded in each lane of the gel for this analysis. std corresponds to recombinant TIMP1 used as a standard control; TE corresponds to whole cell lysate; SE to conditioned medium; and SU to cell surface proteins. B. the secretion rate measurement of TIMP1 and TIMP2 used commercial ELISA kits (R&D systems). Conditioned media were diluted 1:15 and 1:60 for the analysis of TIMP1 and TIMP2, respectively. The secretion rate was estimated based on the slope of the curve at linear time points.

We assessed the enrichment factor for TIMP1 by Western blot analysis (Figure 6a). TIMP1 occurred predominantly in the secreted compartment of all cell lines, consistent with predictions based on spectral counting. We also performed a time-course measurement of TIMP1 and TIMP2 concentration in the conditioned medium to estimate the secretion rate of these proteins for all three cell lines (Figure 6b). TIMP1 was shown to have a low secretion rate in OVCAR3 cells relative to other cell lines, which is consistent with our Western blot analysis. We calculated the secretion rates of TIMP1 and TIMP2 to be on the order of nanograms of protein per million cells, per hour. Based on a secretion rate of 3 ng of TIMP1 per million cells per hour and without taking into account the rate of degradation and clearance in serum, it would take around 10^8^–10^9^ tumor cells and several days of output to alter the levels of TIMP1 that occur in normal human serum, which is 87–524 ng/ml.

## Discussion

We present here an in-depth proteomic analysis of ovarian cancer cell lines and ovarian cancer tumor cells, including separate analyses of cell surface and secreted proteins. The study was based on intact protein fractionation followed by trypsin and LC-MS/MS [16], [33], allowing sensitive detection of low abundance proteins. Altogether, we generated an extensive profile of proteins consisting of more than 6,500 unique identifications with an error rate less than 1%. A recent study of ascites fluid consisting of proteins derived from mixed populations of cells [12] identified 2,737 proteins. 2,307 (84%) of the proteins reported in that study were encompassed among proteins identified in our study.

The use of spectral counts as a measure of abundance coupled with comparisons of proteins found in the cell surface or extracellular compartments and in whole cell lysate profiles, allowed identification of proteins enriched in particular compartments. Peptide counting provides abundance estimates that correlate reasonably well with those determined by other methods [34]. In our study we also observed a good correlation between estimates of abundance of tissue inhibitor of metalloproteinase 1 (TIMP1) and epithelial cadherin (CDH1) based on spectral counting and assessment based on Western blot analysis. Isotopic labeling with SILAC [15] also provided an efficient means to distinguish proteins in conditioned media that are released from cultured cells from proteins contributed by bovine serum in the culture medium.

Our analyses have provided insight into the contribution of surface protein shedding and release to the protein composition of the extra-cellular compartment. Proteins highly enriched in culture media represent a potential source of circulating markers for ovarian cancer. Transcripts corresponding to some of these proteins (e.g., KLK6, 7 and 9) are relatively abundant in ovarian tumors compared to most normal tissues [35]. There is already evidence for the occurrence of most of these proteins in normal human plasma. Of the 60 proteins listed in Table S2, 48 (80%) were present in the Human Plasma Peptide Atlas [26]. However, to be effectively detected at increased levels and as expected of a biomarker candidate, a protein should have high secretion rates specifically by tumor cells, low concentration in normal plasma and also a low clearance from the circulation. Proteins that we identified in the conditioned medium (Table S2) whose corresponding mRNAs are relatively highly expressed in ovarian cancer tissue include TIMP1, IGFBP3 (insulin-like growth factor-binding protein 3), MDK (midkine), PROS1 (vitamin k-dependent protein s) and SLPI (secretory leukoprotease inhibitor). Several other proteins that have been proposed as potential biomarkers for ovarian cancer were identified in the extracellular compartment of cells analyzed in this study, including WFDC2 (HE4) and MUC16 (mucin 16, CA125), a membrane glycoprotein that was significantly enriched in conditioned media from CaOV3, OVCAR3 and ascites cancer cells and which is used as a marker of ovarian cancer [36]. These findings suggest that other proteins enriched in the culture media and/or on the cell surface may have potential as markers for ovarian cancer.

We observed a substantial enrichment of CDH1 both on the cell surface and in culture media, likely as a result of a shedding process driven by metalloproteases [5]. Shed extracellular domains of proteins have potential utility as circulating biomarkers, as has been suggested for some cadherins [37] . Other soluble fragments of membrane proteins, which we found in the extracellular compartment, notably MSLN (mesothelin) have also been proposed as potential biomarkers [38].

The process of surface protein shedding as observed in ascites derived tumor cells is relevant to tumor invasion and metastasis [3], as exemplified by the role of cadherins. Members of this family involved in adherent junctions and desmossomes include desmogleins (DSG1 and DSG2) and the recently discovered family of calystenins (CLSTN1 and CLSTN3). Interestingly, calystenins are localized in the postsynaptic membrane and proteolytically cleaved N-terminal fragments corresponding to the extracellular domain have been observed. Proteolytic cleavage of the extracellular domain of calystenins is known to impact directly Ca2+ signaling in brain [39].

The dynamics of shedding of cell surface proteins and release into circulation (or conditioned media as detected here) has relevance to diagnostics and imaging. However their reduced representation on the cell surface and occurrence in circulation diminishes their utility as imaging or therapeutic targets compared to stable, non-shed proteins. For example, integrin alpha 6 (ITGA6) is significantly enriched on the cell surface of all three ovarian cell lines as well as the ascites tumor cells, is relatively highly expressed in ovarian cancer and was not detected in conditioned media (see Table S4).

Our findings indicate that the serous ovarian cancer derived cell lines OVCAR3 and CaOV3 are good models of serous ovarian cancer to identify potential biomarkers given the similarities we have observed with tumor cells isolated from ascites from a patient with serous ovarian cancer. Our data also indicates extensive cell surface protein shedding in tumor cells obtained from ascites. The extensive proteomic profiling of key compartments for the four ovarian cancer cell populations that we have studies provides a rich dataset for further exploration and integration with other data for identification of potential circulating markers and imaging and therapeutic targets.

## Supporting Information

Figure S1 Unsupervised hierarchical clustering of total peptide counts in all three fractions of the three cell lines and the ascites sample.
(0.03 MB DOC)

Table S1 Proteomic analysis of ovarian cancer cell lines and tumor cells purified from ascites fluid.
(1.75 MB XLS)

Table S2 Proteins with high enrichment in the conditioned media of ovarian cancer cells.
(0.05 MB XLS)

Table S3 Proteins enriched exclusively in tumor cells from ascites.
(0.05 MB XLS)

Table S4 Membrane proteins enriched in both cell surface and culture media or exclusively in cell surface.
(0.07 MB XLS)

## References

[1] Jemal A, Siegel R, Ward E, Murray T, Xu J (2007). Cancer statistics, 2007.. CA Cancer J Clin.

[2] Aletti GD, Gallenberg MM, Cliby WA, Jatoi A, Hartmann LC (2007). Current management strategies for ovarian cancer.. Mayo Clin Proc.

[3] Naora H, Montell DJ (2005). Ovarian cancer metastasis: integrating insights from disparate model organisms.. Nat Rev Cancer.

[4] Rosso O, Piazza T, Bongarzone I, Rossello A, Mezzanzanica D (2007). The ALCAM shedding by the metalloprotease ADAM17/TACE is involved in motility of ovarian carcinoma cells.. Mol Cancer Res.

[5] Symowicz J, Adley BP, Gleason KJ, Johnson JJ, Ghosh S (2007). Engagement of collagen-binding integrins promotes matrix metalloproteinase-9-dependent E-cadherin ectodomain shedding in ovarian carcinoma cells.. Cancer Res.

[6] Hanash SM, Pitteri SJ, Faca VM (2008). Mining the plasma proteome for cancer biomarkers.. Nature.

[7] An HJ, Kim DS, Park YK, Kim SK, Choi YP (2006). Comparative proteomics of ovarian epithelial tumors.. J Proteome Res.

[8] Bengtsson S, Krogh M, Szigyarto CA, Uhlen M, Schedvins K (2007). Large-scale proteomics analysis of human ovarian cancer for biomarkers.. J Proteome Res.

[9] Gagne JP, Ethier C, Gagne P, Mercier G, Bonicalzi ME (2007). Comparative proteome analysis of human epithelial ovarian cancer.. Proteome Sci.

[10] Morita A, Miyagi E, Yasumitsu H, Kawasaki H, Hirano H (2006). Proteomic search for potential diagnostic markers and therapeutic targets for ovarian clear cell adenocarcinoma.. Proteomics.

[11] Young TW, Rosen DG, Mei FC, Li N, Liu J (2007). Up-regulation of tumor susceptibility gene 101 conveys poor prognosis through suppression of p21 expression in ovarian cancer.. Clin Cancer Res.

[12] Gortzak-Uzan L, Ignatchenko A, Evangelou AI, Agochiya M, Brown KA (2008). A proteome resource of ovarian cancer ascites: integrated proteomic and bioinformatic analyses to identify putative biomarkers.. J Proteome Res.

[13] Lin YW, Lin CY, Lai HC, Chiou JY, Chang CC (2006). Plasma proteomic pattern as biomarkers for ovarian cancer.. Int J Gynecol Cancer.

[14] Moshkovskii SA, Serebryakova MV, Kuteykin-Teplyakov KB, Tikhonova OV, Goufman EI (2005). Ovarian cancer marker of 11.7 kDa detected by proteomics is a serum amyloid A1.. Proteomics.

[15] Ong SE, Mann M (2006). A practical recipe for stable isotope labeling by amino acids in cell culture (SILAC).. Nat Protoc.

[16] Faca V, Pitteri SJ, Newcomb L, Glukhova V, Phanstiel D (2007). Contribution of protein fractionation to depth of analysis of the serum and plasma proteomes.. J Proteome Res.

[17] Rauch A, Bellew M, Eng J, Fitzgibbon M, Holzman T (2006). Computational Proteomics Analysis System (CPAS): an extensible, open-source analytic system for evaluating and publishing proteomic data and high throughput biological experiments.. J Proteome Res.

[18] Keller A, Nesvizhskii AI, Kolker E, Aebersold R (2002). Empirical statistical model to estimate the accuracy of peptide identifications made by MS/MS and database search.. Anal Chem.

[19] Nesvizhskii AI, Keller A, Kolker E, Aebersold R (2003). A statistical model for identifying proteins by tandem mass spectrometry.. Anal Chem.

[20] Choi H, Nesvizhskii AI (2008). False discovery rates and related statistical concepts in mass spectrometry-based proteomics.. J Proteome Res.

[21] Liu H, Sadygov RG, Yates JR (2004). A model for random sampling and estimation of relative protein abundance in shotgun proteomics.. Anal Chem.

[22] Kall L, Krogh A, Sonnhammer EL (2007). Advantages of combined transmembrane topology and signal peptide prediction–the Phobius web server.. Nucleic Acids Res.

[23] Emanuelsson O, Brunak S, von Heijne G, Nielsen H (2007). Locating proteins in the cell using TargetP, SignalP and related tools.. Nat Protoc.

[24] Kaiser BK, Yim D, Chow IT, Gonzalez S, Dai Z (2007). Disulphide-isomerase-enabled shedding of tumour-associated NKG2D ligands.. Nature.

[25] Shin BK, Wang H, Yim AM, Le Naour F, Brichory F (2003). Global profiling of the cell surface proteome of cancer cells uncovers an abundance of proteins with chaperone function.. J Biol Chem.

[26] Desiere F, Deutsch EW, King NL, Nesvizhskii AI, Mallick P (2006). The PeptideAtlas project.. Nucleic Acids Res.

[27] Hamilton TC, Young RC, McKoy WM, Grotzinger KR, Green JA (1983). Characterization of a human ovarian carcinoma cell line (NIH:OVCAR-3) with androgen and estrogen receptors.. Cancer Res.

[28] Karlan BY, Jones J, Slamon DJ, Lagasse LD (1994). Glucocorticoids stabilize HER-2/neu messenger RNA in human epithelial ovarian carcinoma cells.. Gynecol Oncol.

[29] Lau DH, Lewis AD, Ehsan MN, Sikic BI (1991). Multifactorial mechanisms associated with broad cross-resistance of ovarian carcinoma cells selected by cyanomorpholino doxorubicin.. Cancer Res.

[30] Shaw JL, Diamandis EP (2007). Distribution of 15 human kallikreins in tissues and biological fluids.. Clin Chem.

[31] Su AI, Wiltshire T, Batalov S, Lapp H, Ching KA (2004). A gene atlas of the mouse and human protein-encoding transcriptomes.. Proc Natl Acad Sci U S A.

[32] Maatta M, Talvensaari-Mattila A, Turpeenniemi-Hujanen T, Santala M (2007). Matrix metalloproteinase-2 (MMP-2) and -9 (MMP-9) and their tissue inhibitors (TIMP-1 and TIMP-2) in differential diagnosis between low malignant potential (LMP) and malignant ovarian tumours.. Anticancer Res.

[33] Faca V, Krasnoselsky A, Hanash S (2007). Innovative proteomic approaches for cancer biomarker discovery.. Biotechniques.

[34] Gilchrist A, Au CE, Hiding J, Bell AW, Fernandez-Rodriguez J (2006). Quantitative proteomics analysis of the secretory pathway.. Cell.

[35] Schwartz DR, Kardia SL, Shedden KA, Kuick R, Michailidis G (2002). Gene expression in ovarian cancer reflects both morphology and biological behavior, distinguishing clear cell from other poor-prognosis ovarian carcinomas.. Cancer Res.

[36] Goonewardene TI, Hall MR, Rustin GJ (2007). Management of asymptomatic patients on follow-up for ovarian cancer with rising CA-125 concentrations.. Lancet Oncol.

[37] De Wever O, Derycke L, Hendrix A, De Meerleer G, Godeau F (2007). Soluble cadherins as cancer biomarkers.. Clin Exp Metastasis.

[38] Scholler N, Fu N, Yang Y, Ye Z, Goodman GE (1999). Soluble member(s) of the mesothelin/megakaryocyte potentiating factor family are detectable in sera from patients with ovarian carcinoma.. Proc Natl Acad Sci U S A.

[39] Vogt L, Schrimpf SP, Meskenaite V, Frischknecht R, Kinter J (2001). Calsyntenin-1, a proteolytically processed postsynaptic membrane protein with a cytoplasmic calcium-binding domain.. Mol Cell Neurosci.

