# Supplementary material for: Proteomic Analysis of Ovarian Cancer Cells Reveals Dynamic Processes of Protein Secretion and Shedding of Extra-Cellular Domains
Source: PLoS One. 2008 Jun 18;3(6):e2425. doi: 10.1371/journal.pone.0002425 (PMC2409963; doi:10.1371/journal.pone.0002425)

**Figure S1. Unsupervised hierarchical clustering of total peptide counts in all three fractions of the three cell lines and the ascites sample.** The analysis it was performed with a centroid similarity metric and centroid linkage using the Cluster software (Eisen et al. 1998, PNAS 95: 14863-14868). “Asc” stands for cancer cells enriched from ascites in the diagram.


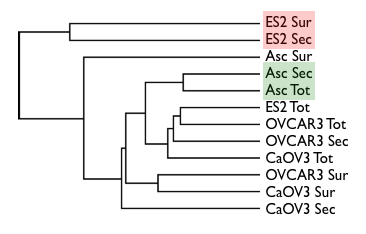

Supplement: Figure S1 — Unsupervised hierarchical clustering of total peptide counts in all three fractions of the three cell lines and the ascites sample. (0.03 MB DOC) [file pone.0002425.s001.doc]
